# Our Contemporaries

**Published:** 1913-01

**Authors:** 


### OUR CONTEMPORARIES.

---

*Our Dumb Animals,* December, 1912, approves our editorial suggestion as to cooperation to secure a rational vivisection bill but objects to the insistence of professional *control.* It is necessary in any moot subject that the final decision shall rest with the party having the most expert knowledge of the problem and it is not immodest, in this case, to claim control for the medical profession. Two facts are overlooked by many antivivisectionists: most physiologic experimenters are humane men; painful experiments introduce a disturbing reflex factor capable, for instance, of suspending secretion, of raising blood pressure and, in various other ways, of vitiating the result of experiment. 99 per cent of all painful experiments are foolish. The association of cruelty and piety is a curious one. In an old book, we read of a cruel experiment: a pregnant dog is delivered by Caesarian section to show—not a physiologic fact to physiologists, nor a surgical procedure that might help surgeons to save human life—but the dominance of maternal affection, the parent licking her offspring in spite of her pain; and this cruel and unnecessary experiment was performed for the edification of theologians. A few years ago, a gynaecologist in Germany suggested the acquisition of surgical skill in hebosteotomy by operating on the sheep, just before it gets the coup de grace at a slaughter house: and we donounced the cruelty of this suggestion in the *Medical Times,* pointing out that both mercy and operative analogy demanded that the practice operation be performed after the stunning or killing of the animal. At about the same time, some one reported to a medical or surgical association, a series of cruel mutilation of rats to demonstrate the existence of a "sixth" sense, and the paper was denounced by the profession as indicative both of cruelty and an unpractical, metaphysical conception at variance with the pragmatism of modern medical science.

---

*Life* has devoted a recent issue to the Doctor. The issue is of real value in determining the ethical standing of a few advertisements that have been carried by some medical journals and, negatively, so far as such evidence goes, of some others that have been criticised in certain quarters but which are restricted to medical journals. We regret that so many pages have been devoted to jests about the avarice, brutality and conceit of a profession which is, on the whole, poorly paid and almost without limit in its charity; which, so to speak, makes a business of being gentle and kind; and which realizes its lack of definite knowledge in many ways, and to such a degree that many of its members spend almost their entire leisure and nearly every cent of their income beyond a modest living, in trying to obtain more knowledge. Moreover, in every average issue, we note the death of some doctor, not as the result of the ordinary strain and risk of his work, but as a definite martyrdom in the attempt to save a particular life, or to advance medical knowledge in general. As we write, our attention is called to a news notice of three railway surgeons risking their own lives, not in any strictly professional work that might be ascribed to habit, or in which the cynic might ascribe the risk to hope of glory, but in the hard manual task performed in the cold and dark, of sawing a railroad victim, out of a steel car. But, on the whole, *Life's* doctors' number is too hackneyed to call for either a laugh or a tear.

---

A VACCINATION *YARN*. *Life,* Nov. 7,1912, quotes from *Open Door* "this sunny little anecdote of a school girl in Buffalo." "When two public vaccinators and two policemen visited her school to inoculate the pupils with virus at $1.00 each, nine-year-old Lucille objected, saying she had been vaccinated and, if it must be done again, she would go home to have it done. But she was threatened and force was used. Thirteen days later and after ten days of suffering from blood poisoning, she died. Her mother lost her reason and was put in an asylum."

Inaccuracy No. 1. Vaccination in the schools of Buffalo is free to pupils. The vaccinators are not paid a dollar nor any other sum per capita, but perform this service, as part of their duty as city physicians. They are employed on an annual salary, or rather wage, as it is less than the average of clerical positions of low scale.

Inaccuracy No. 2. Every pupil is given an opportunity to be vaccinated by a physician privately employed and the certificate of any qualified physician is honored.

Inaccuracy No. 3. The references to threats and force are misleading. The law requires vaccination and it is obvious that nervous children or those encouraged by parents to resist the law may require control by methods which the prejudiced might thus describe, just as school discipline in any other respect might require coercion.

As to the gist of the matter, after careful inquiry of the Health and Educational authorities, we find the following as the only tangible basis for the story: A few years prior to 1900, a girl aged about five, in the first year of the grammar school, died after vaccination. There was considerable doubt as to the exact cause of death but an expert gave his opinion that it was due to vaccinia in virulent form. It is well known that vaccinia itself does, very rarely, assume this type and that it may be fatal. So far as we can learn, this is the only case of death resulting from vaccination that has occurred in the city of Buffalo since records have been kept. However much we may regret such an occurence, it cannot be ascribed to carelessness; it is the inevitable risk of one in several hundred thousand cases necessary to protect against the almost inevitable occurrence of small pox on a large scale and the approximately one chance in ten that each case would die. It is one death in, say thirty years, as opposed to an average of about 25 deaths a year from scarlet fever—a fair comparison, as without vaccination, the two diseases are about equally prevalent and fatal.

We cannot learn that any child's mother ever became insane in Buffalo, because of vaccination.

If our statements are wrong, in any respect, we will publish corrections.

---

The *International Hospital Record,* Nov., 1912, in an editorial on the prevention of overstrain in nurses, regards actual overwork as a minor factor and mentions the necessity of overseeing the dietetic habits, nagging by head nurses, insufficient out-door recreation, etc. To which we may add the constant fear of discharge for minor infractions of rules, and the exercise of discipline and interference with personal liberty not encountered by any other class of students.

The same journal quotes, with approval, our criticism on the Baltimore resolution aimed at the suppression of news items mentioning patients and hospitals and adds:

"It may be well for those who seek to make the medical profession a 'close corporation' to bear in mind that the general public may come to the point where it will express itself regarding this. The recent national elections shows that the general public has developed of late years a tendency to speak out with remarkable force. The medical profession is entitled to all the honor it deserves by virtue of efficiency and broad-mindedness, and it should guard carefully against any action such as may be construed as narrow and selfish."

---

The *Bulletin of the Johns Hopkins Hospital,* Dec., 1912, contains an interesting historic article on the Last Illness of Louis XIV of France, by Dr. Carroll E. Edson of Denver.

---

*Puck* published the following which is appropriate to the medical profession, though not so designated: "The gods, perceiving that man was of few days and full of trouble, gave him philosophy. But, at that, the fellow was more comfortable than he had any right to be. 'Give him ethics,' the gods thereupon directed, by way of evening things up."

---

*Pearson's Magazine,* for January contains an article on the Schafer Phylacogens, by Arno Dosch. We question seriously whether the dissemination of rudimentary information in such detail is wise. The publication of such an article also places the manufacturers of a medicinal preparation in an unpleasant light. In the present case, as in another recently mentioned, the manufacturers are absolutely blameless. Messrs. Parke, Davis & Co. have submitted copies of five letters passed between their office and that of *Pearson's Magazine,* covering the period from Oct. 17 to Oct. 25, showing that the firm made a vigorous protest against the publication of the article both on ethical grounds and on the practical objection that an imperfect popular conception of a remedial agent would lead to unwise attempts at its use and create an exaggerated opinion of its efficacy. The matter was left with the understanding that the magazine would abide by the decision of a number of reputable physicians, which it would choose—with the obvious result.

---

THE AMERICAN MEDICAL ASSOCIATION has appointed a committee to correspond with the British Medical Association looking to a joint meeting of the two bodies. The committee is composed of: Drs. A. T. Bristow, of New York; W. W. Grant, of Denver; G. H. Simmons, Chicago; T. W. Huntington, San Francisco, and E. J. Goodwin, St. Louis.

---

*The Homoeopathic Recorder,* Dec., 1912, approves the spirit of our remarks as to medical unity in so far as harmony and friendship are concerned but doubts the possibility of *unity* in the strict sense, on the ground that opposites and similars cannot mingle. When "old-school" physicians attend the annual ball of the Homoeopathic Hospital; when the Homoeopathic Hospital Staff sends a beautiful bouquet of roses to grace the opening festivities of the German Deaconess Hospital, that is harmony. But when Homoeopathic physicians frequent the Academy of Medicine, read and discuss papers; when "regulars" take patients to the Homoeopathic Hospital and find internes secretly smiling at their small doses; that comes pretty close to practical unity. Remember, very few of us hold to the doctrines of opposites. That is why we are so touchy about being called Allapaths, not because we want to claim the term "regular" in an autocratic sense. In fact, we don't want any specific designation beyond that of our profession. There is unity, as well as harmony between friends who call each other—quite in a friendly way—all sorts of hard names over questions of operation and medication, of abdominal and vaginal routes, of choice of antiseptics, heart tonics, etc. We want and in western New York, we have pretty nearly attained, the same kind of unity across what used to be hard lines of school opinion.

The Denver Medical Times and Utah Medical Journal has been further enlarged to include a Nevada section. Each section is independently edited by Dr. Edward C. Hill of Denver, Dr. Frederic Clift of Layton, Utah, and Dr. George L. Servoss of Gardnerville, Nev. Jno. A. Stanson, the Business Manager, contributes to the Dec. issue, a scathing editorial "So that the perfect one may know," which we consider of rather doubtful value to the cause of independent journalism. Colorado, Utah and Nevada is a wide field for a local journal yet, in population, Western New York from Buffalo to Utica, is equivalent to these three states and also Wyoming, Montana, N. Dakota and S. Dakota.

